# Evaluation of Thermal and Thermomechanical Behaviour of Bio-Based Polyamide 11 Based Composites Reinforced with Lignocellulosic Fibres

**DOI:** 10.3390/polym9100522

**Published:** 2017-10-18

**Authors:** Helena Oliver-Ortega, José Alberto Méndez, Pere Mutjé, Quim Tarrés, Francesc Xavier Espinach, Mònica Ardanuy

**Affiliations:** 1 Group LEPAMAP, Department of Chemical Engineering, University of Girona, C/M.Aurèlia Capmany, 61, 17003 Girona, Spain; helena.oliver@udg.edu (H.O.-O.); jalberto.mendez@udg.edu (J.A.M.); pere.mutje@udg.edu (P.M.); 2 Design, Development and Product Innovation, Dpt. Organization, Business Management and Product Design, University of Girona, C/M.Aurèlia Capmany, 61, 17003 Girona, Spain; francisco.espinach@udg.edu; 3 Departament de Ciència dels Materials i Enginyeria Metal.lúrgica, Secció Enginyeria Tèxtil, Universitat Politècnica de Catalunya, C/Colom, 11, 08222 Terrassa, Barcelona, Spain; monica.ardanuy@upc.edu

**Keywords:** polyamide 11, lignocellulosic fibres, thermomechanical behaviour, annealing, microstructure

## Abstract

In this work, polyamide 11 (PA11) and stone ground wood fibres (SGW) were used, as an alternative to non-bio-based polymer matrices and reinforcements, to obtain short fibre reinforced composites. The impact of the reinforcement on the thermal degradation, thermal transitions and microstructure of PA11-based composites were studied. Natural fibres have lower degradation temperatures than PA11, thus, composites showed lower onset degradation temperatures than PA11, as well. The thermal transition and the semi-crystalline structure of the composites were similar to PA11. On the other hand, when SGW was submitted to an annealing treatment, the composites prepared with these fibres increased its crystallinity, with increasing fibre contents, compared to PA11. The differences between the glass transition temperatures of annealed and untreated composites decreased with the fibre contents. Thus, the fibres had a higher impact in the composites mechanical behaviour than on the mobility of the amorphous phase. The crystalline structure of PA11 and PA11-SGW composites, after annealing, was transformed to α’ more stable phase, without any negative impact on the properties of the fibres.

## 1. Introduction

Composites are produced to obtain new materials, designed to be used for a specific applications, with comparatively better properties than its phases [1]. In this sense, the literature shows that polymeric matrices with comparatively poor mechanical properties, such as polyolefin, have been successfully reinforced to obtain competitive materials. One example of these composites are glass fibre reinforced polypropylene (PP) materials, currently produced and used at industrial level [2]. Nonetheless, despite the high mechanical performance of these composites, there are health problems associated with the manipulation of glass fibres [3] and its poor recyclability. This justifies the search for more healthy and environmentally friend substitutive reinforcements. Moreover, the UE has fixed for 2025–2030 some recyclability goals [4] which are not possible to achieve using glass fibres as reinforcements. 

Cellulosic or lignocellulosic reinforcing fibres have become a recyclable alternative to glass fibres. These fibres have been extensively studied since the 1980s showing good intrinsic properties such as comparatively high tensile strengths [5]. Moreover, cellulosic fibres are fully bio-based and biodegradable [6]. Besides, the use of natural fibres accomplishes objectives proposed by green chemistry and sustainable production [7,8]. In addition, their use and manipulation is healthier. 

Since the 1980s, the environmental awareness of the society has increased noticeably. The use of non-renewable resources and the high impact of oil-based polymers on the environment drive the research of more environmentally friendly alternatives to such resources. One of the proposed challenges is reducing the oil-based polymer dependence and replacing these polymers by bio-based or biodegradable ones [9], with lower environmental fingerprints [10]. Besides, the use of biodegradable polymers can also promote waste reduction, diminishing its environmental impact [11]. Therefore, the composite materials researchers have increased their interest on these materials as possible replacements to oil-based matrices [12]. 

Despite all the aforementioned advantages, cellulosic fibres have low thermal degradation temperatures, being this its main drawback that limits their use as reinforcement for polymer-based composites [12]. The degradation temperatures of cellulosic fibres are between 200 °C and 250 °C, and, therefore, the matrices must be able to be processed at such temperatures. Polyamide 11 (PA11), also called nylon 11, is a bio-based polyamide obtained from castor oil. Moreover, it is a non-biodegradable polymer matrix allowing its use for long-time applications, such as those needed in the construction or automotive sectors [12,13]. Besides, its melting temperature, around 190 °C, is low enough to avoid the thermal degradation of cellulosic fibres during composites manufacturing. Furthermore, PA11 is a recyclable thermoplastic matrix with mechanical properties similar to polypropylene. Thus, PA11 is a promising green alternative to polypropylene [14,15].

There are some studies in the about different nanomaterial reinforced PA11 nanocomposites [16,17]. More recently, there are works devoted to cellulosic fibres reinforced PA11 composites [15,18,19]. The analysis of the tensile and flexural properties of these cellulosic composites showed significant increases of its modulus and strength together with low reductions of its strains at break [15,19], compared to other fibre, like glass fibres (GF), based composites [14]. However, although the thermal properties and the structure of PA11-based composites reinforced with several micro- or nano-reinforcements have been extensively studied, there are only few studies about the effect of cellulosic fibres in a PA11 matrix [15]. 

In this study, Stone Groundwood (SGW) fibres from softwood were used as reinforcement for PA11 biocomposites. SGW fibres were obtained by high yield mechanical processes (98%) and, therefore, the chemical composition of such fibres and the wood from which they come were the same [19]. Moreover, SGW is produced in a sustainable way due to its use in the papermaking industry. The effect of these lignocellulosic fibres in the thermal stability, thermal transitions and thermomechanical behaviour of PA11 composites were analysed. In addition, an annealing treatment was applied to the fibres to study the impact of such treatment in the thermal properties of the composites.

## 2. Materials and Methods

### 2.1. Materials

Rilsan® BMNO TLD polyamide 11, kindly supplied by Arkema S.A. (Colombes, France), with a density of 1.03 g/cm^3^ and a melt flow index (MFI) of 11 cc/10 min measured at 235 °C/2.16 kg, was used as polymer matrix.

Stone Groundwood (SGW), a mechanically defibrated pulp from softwood (*Pinus radiata*), provided by Zubialde S.A. (Aizarnazabal, Spain), was used as reinforcement. The length and diameter distributions of SGW fibres were studied in a previous work [19]. In the mentioned study, the density of fibre was determined to be 1.40 g/cm^3^.

### 2.2. Composite Compounding

Composites reinforced with 10%, 20% and 50% *w*/*w* fibre content were produced in a Gelimat Kinetic Mixer (model G5S, Draiswerke, Mahaw, New Jersey, NJ, USA). The fibres and the polymer matrix were firstly added and premixed at low speed (300 rpm) and then the speed was increased up to 2500 rpm. When the mixture reached 200 °C, the material was discharged, cooled and pelletized with a knives mill. To produce the specimens, the composites were mould injected in a Meteor-40 injection machine (Mateu & Solé, Barcelona, Spain). The processing temperature profile was 170–185–200 °C and the pressures were modified regarding the fibre content to a maximum of 75 bars for the volumetric phase and 30 bars for the pressure maintenance phase. The samples were stored in a climatic chamber at 23 °C and 50% RH before their analysis, according to ASTM D618 standard specifications.

### 2.3. Annealing Treatment

The annealing treatment, based previous publications, was performed following the conditions that led to a maximum crystallinity enhancement [20,21]. This treatment consisted of heating the matrix or the composites in an oven at 165 °C for 1 h followed by cooling them at room temperature (23 °C).

### 2.4. Composite Characterization

Thermogravimetrical analysis (TGA) was performed in a Mettler Toledo SDTA 851 thermobalance (Mettler Toledo, L’Hospitalet de Llobregat, Spain). Samples were heated from 30 to 700 °C at a heating rate of 10 °C/min under nitrogen atmosphere at a flow rate of 40 mL/min. 

Differential Scanning Calorimetry analysis (DSC) was performed using a Mettler Toledo DSC822e calorimeter (Mettler Toledo, L’Hospitalet de Llobregat, Spain) following ASTM E 1269.01 standard specification. The samples were initially heated from 40 to 210 °C to erase their thermal history. Afterwards, the samples were cooled and heated again using the same temperature range. All runs were performed at heating or cooling rates of 10 °C/min under 40 mL/min flow of nitrogen atmosphere.

Dynamic mechanical thermal analysis (DMTA) was carried out in a Mettler Toledo DMA/SDTA 861 (Mettler Toledo, L’Hospitalet de Llobregat, Spain) using dual cantilever configuration. Specimens of 65 × 13 × 3 mm^3^ were cut from the flexural specimens obtained following the ASTM D3641. The tests were performed at a frequency of 1 Hz and a preload of 3 N. The temperature range was −40 °C to 120 °C with a heating rate of 3 °C/min and the analysis was performed in air atmosphere. 

X-ray diffraction measurements were performed on a Bruker D8 Advance diffractometer (Bruker, Madrid, Spain) with a Cu-Kα radiation (λ = 0.15406 nm). Data were collected on the 2θ range from 5° to 40° operating at 40 KV and 40 mA. 

A Fourier Transformed infrared spectroscopy (FT-IR) using a Bruker Alpha FT-IR spectrometer (FT-IR) (Bruker, Madrid, Spain) was performed in the PA11 and PA11 composites. 

## 3. Results and Discussion

### 3.1. Thermal Stability of the Composites

As mentioned in the Introduction, the comparatively low degradation temperature of natural fibres limits its use with a broad set of polymeric matrices. The matrices must have melting temperatures in the order of 200 °C. Moreover, the fibre degradation could have a negative effect on the thermal degradation of the polymer matrix. 

**Figure 1:**
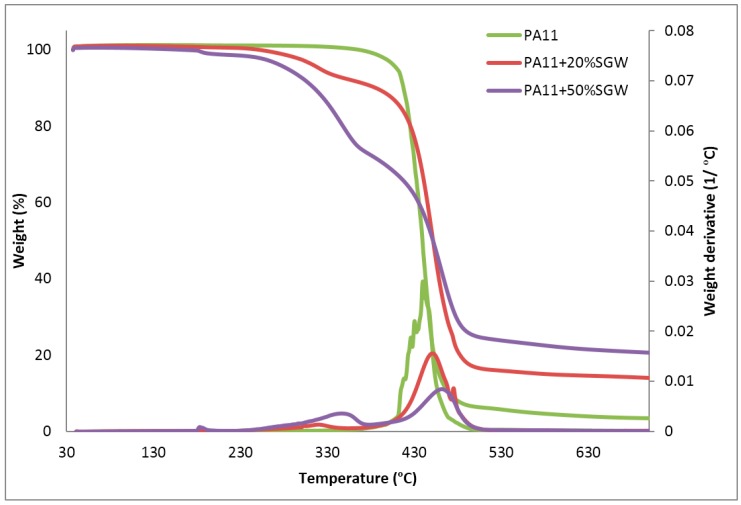
TGA curves and first derivate of the TGA curve for neat PA11 and PA11-SGW composites.

The TGA profiles and the first derivate of the PA11 and the composites reinforced with a 20% and 50% *w*/*w* of SGW are shown in Figure 1.

As shown in Figure 1, neat PA11 presents a TGA trend with one main decomposition step starting around 400 °C. PA11 composites started degrading before, and presented two main decomposition steps, as was expected for cellulose reinforced composite materials. Table 1 shows the onset temperatures for the 5% and 10% of weight loss (*T*_5%_ and *T*_10%_) in PA11 and PA11-SGW composites. 

Although the degradation of PA11-SGW composites seemed to start around 200 °C, the weight loss did not surpass 5% until 300 °C. This first decomposition step was related to the reinforcement degradation, and gained importance when the fibre contents were increased. During this step, the degradation of the O-glucosidic bonds of the cellulose and hemicelluloses occurred [22,23]. The degradation of the other component of the fibres, lignin and extractives occurred in a broader temperature range from around 200 °C to 900 °C [24,25]. SGW fibres showed high lignin contents [19,26], thus, a high degradation range was expected. It was considered that the slight differences obtained in the fibres length, produced by attrition phenomena during composite preparation of the fibres [14,19], had little influence in its decomposition. Moreover, it was possible to measure the onset temperature for the 95% weight loss (*T*_95%_) for the matrix (560 °C) but it was impossible for the composites. An inflection point in the curve, where a second degradation step started, was observed at around 350 °C and 375 °C for PA11 + 20% SGW and PA11 + 50% SGW, respectively. This was related with the degradation of the polymeric phase. This inflection point in the curve indicated that the matrix started degrading before the fibre degradation was finished [22]. The first derivative of the TGA curve was performed to evaluate this effect (Figure 1). As expected, only one peak was obtained for the PA11 matrix, while two peaks, corresponding with the degradation of the fibres and polymer matrix, were found in PA11-SGW composites. Moreover, these peaks appeared overlapped indicating that there was a range of temperatures where fibres and matrix degraded simultaneously.

On the other hand, a shift of the maximum temperature of the decomposition step (*T*_max_) of the polymer from 439 °C for pure PA11 to 451 °C and 461 °C for PA11 + 20% SGW and PA11 + 50% SGW composites, respectively, was observed. From these results, it was concluded that, although the cellulose fibres had a negative effect on the onset decomposition temperature of the composites, their presence contributed to thermally stabilize the composite once the degradation started. The literature shows similar thermal stabilizations in the case of cellulose nanofiber (CNF) reinforced PA11 [27] and other thermoplastic matrices reinforced with lignocellulosic fibres [28]. This phenomenon has been explained as an inhibiting effect of the char obtained from the fibres decomposition to the diffusion of volatile and radical compounds implied in PA11 decomposition. 

Another important effect of the fibre addition was related to the residue found at 700 °C. It was observed that the addition of fibre enhanced its content from 3.4%, obtained in monolithic PA11, up to 20.5% when 50% SGW fibres were added to the composite material. This increase on the residue was related with the extractives and inorganic compounds contents in the lignocellulosic reinforcement, and lignin molecules which did not totally degrade [24,25,29]. Finally, from these TGA curves, it was observed that the degradation of the fibres occurred at higher temperatures than the processing temperatures of the corresponding composites, allowing the preparation of these composites.

### 3.2. Thermal Transitions and Structure of the Composites

**Figure 2:**
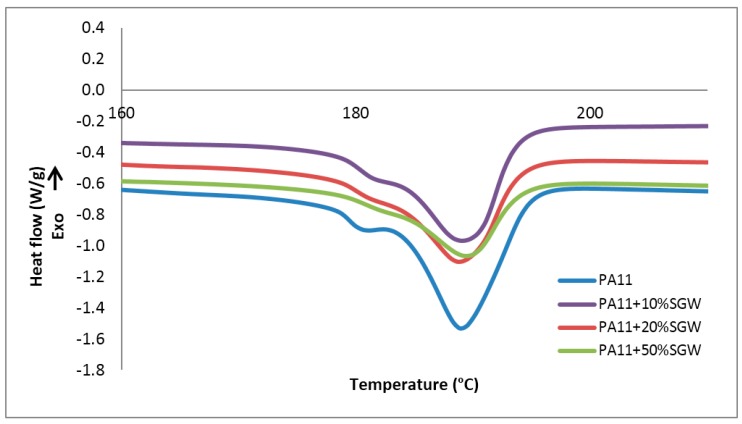
DSC thermographs of the melting point of PA11 and PA11 composites.

The melting and crystallization behaviours of the polymer matrix and the composites were studied by means of DSC. The thermographs obtained during the second heating, after erasing the thermal history, are shown in Figure 2. 

It was observed that PA11 presented a main peak at around 189 °C, preceded by a shoulder with a maximum at around 181 °C as a rearrangement in the structure [30]. PA11 has different crystalline structures which can be transformed from one to another depending on temperature, cooling conditions and pressure, among others [31,32,33]. This shoulder peak corresponded to the melt-crystallization process of the γ phase to the α’ crystalline form of PA11 [30,34]. The secondary peak seemed to decrease and the main peak became broader when the fibre content was increased in the composite material. This effect has also been found when CNF were used as reinforcement, indicating that the fibres promoted the α’ crystalline form instead of the γ form observed in the neat PA11 [27,34]. On the other hand, it was found that the melting temperature (*T*_m_) was not affected by SGW presence (189 °C for all the studied materials). 

The degree of crystallinity of the polymer matrix was calculated as the ratio between the enthalpy of main melting endotherm and the theoretical enthalpy for a fully crystalline polymer matrix ($\Delta \hat{H}$m = 226.4 J/g) [27,35]. The obtained degree of crystallinities for the composites materials were 26.4%, 26.4% and 27.2% for PA11 + 10% SGW, PA11 + 20% SGW and PA11 + 50% SGW, respectively. These values were very similar to the pure matrix (26.7%), indicating no significant effect of the fibres on the degree of crystallinity of the PA11 matrix. In the literature, a slight increase (around 3%) in the degree of crystallinity for CNF reinforced PA11 composites with contents lower than 5% has been reported [27]. However, for higher contents of CNF, the crystallinity decreased. This could be related with a disruption of the PA11 structure by the effect of the CNF [27]. Moreover, the chemical surface’s composition of SGW fibres is different to CNF studied in the literature, mainly due to the presence of lignin in the fibre surface. This could inhibit the nucleating effect of the fibres, as it also inhibited the interactions between the polymer matrix and the fibre [15,19,29]. On the other hand, cellulose nanocrystals (CNC) and modified CNC also obtained no significant changes in crystallinity when they were used as reinforcement in PA11 [36]. This is the opposite effect to that observed in other polymer matrices, where the cellulosic fibres acted as nucleating agents, increasing the polymer crystallinity [22,37,38]. Nonetheless, these matrices had weaker interactions with cellulose fibres than PA11. The capacity of this polymer to establish H-bonds with cellulose [19] can explain this disruption in the polymer matrix, and hence no considerable nucleating effect was observed. Moreover, this capacity can inhibit the formation of the γ crystalline phase on the composites. 

Concerning the crystallization behaviour, a peak with a maximum crystallization temperature (*T*_c_) at 164 °C was observed for the PA11 and the composites. The crystallinity of PA11 matrix was also measured as the ratio between the enthalpy of the polymer crystallization and the theoretical value of the crystalline polymer matrix. The obtained values were not different from those obtained during the melting except for the PA11 + 10% SGW composite, where a lower degree of crystallinity (22.8%) was measured. 

The results allowed concluding that, as found in the literature for other cellulose reinforced PA11 and PP composites, the presence of SGW fibres did not affect the main transition temperatures of the crystalline polymer phase [22,33,37].

The DMTA thermograms of the PA11 composite materials was performed to observe other processes in which the material loss energy take place, as well as to understand the behaviour of the stiffness of the materials with the temperature. In this sense, a softening is usually experimented when thermoplastic materials overpass the glass transition (*T*_g_) as the amorphous chains of the polymer suffers an important gain of mobility.

**Figure 3:**
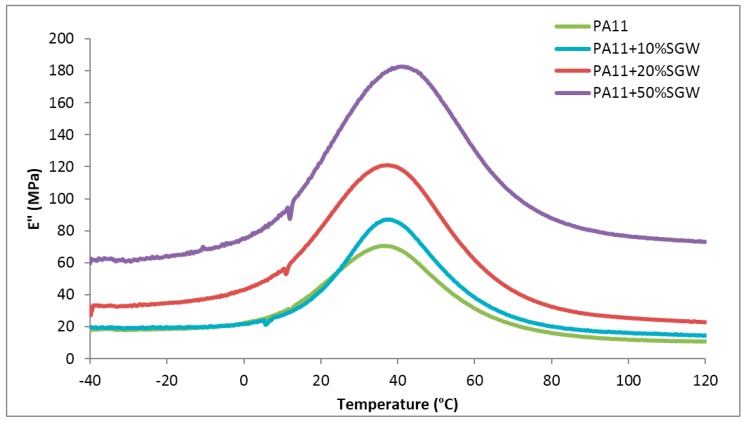
Loss Modulus of the PA11, PA11 + 20% SGW and PA11 + 50% SGW with respect to the temperature.

As shown in Figure 3, the evolution of the loss modulus (E”) of pure PA11 and the composites with respect to the temperature showed a unique transition in the studied range, related with the *T*_g_ of the polymer matrix. 

**Figure 4:**
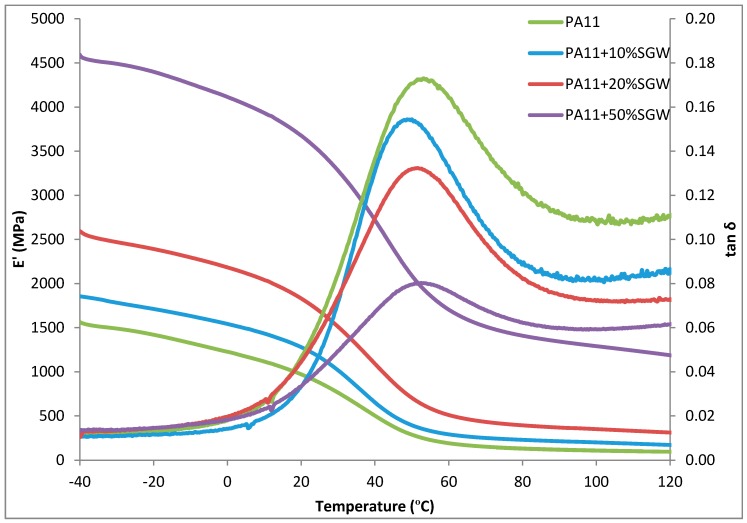
Storage modulus and tan δ results of PA11, PA11 + 10% SGW, PA11 + 20% SGW and PA11 + 50% SGW.

Figure 4 represents the evolution of the values of storage modulus (E’) and tan δ of PA11 and the composites with the temperature. The measured values of the *T*_g_ obtained from the tan δ curve shifted from 53.1 °C for neat PA11 °C to 50.0 °C, 51.0 °C and 53.2 °C for the PA11 + 10% SGW, PA11 + 20% SGW and PA11 + 50% SGW, respectively. No considerable differences were observed in the *T*_g_ of the composites by the effect of SGW content. This was related with the same crystallinity values of the materials. Thus, no changes were observed in the amorphous phase of the PA11 matrix in the composite materials. It was observed that tan δ decreased with SGW contents as a consequence of the enhancement of the loss moduli.

As expected, higher values of storage moduli were obtained for the composite materials due to the stiffening effect of the reinforcing material [39,40]. For all materials, a slight decrease was observed when the temperature was raised in the analysis below 20 °C, as a result of a slight mobility gain in the polymer chain, and a drastic drop was observed when the *T*_g_ was overpass. Once the temperature was over the *T*_g_, the storage modulus values were really low, indicating a high mobility of the polymer molecules corresponding to the amorphous phase of the PA11. However, the presence of SGW fibres stiffened the material, achieving higher values of storage modulus due to the higher stiffness of cellulosic fibres and counteracting the reduction of the modulus when the *T*_g_ was exceeded. It must be noted that the presence of lignocellulosic fibres in the composite material significantly reduced the mobility of the polymer chains for temperatures higher than its *T*_g_. The influence of SGW fibres is clearly observed in Figure 4, where the modulus of PA11 + 50% SGW at 80 °C is 10 times higher than the PA11 modulus at the same temperature and slightly higher than the PA11 matrix at 20 °C. 

**Figure 5:**
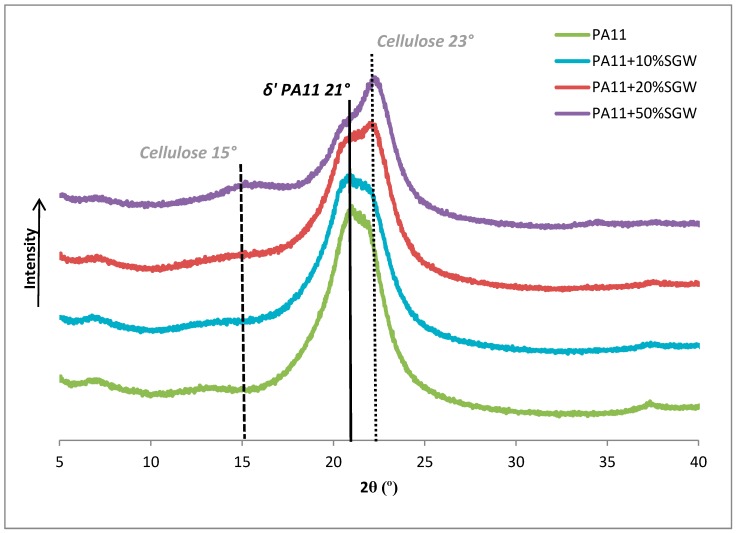
X-ray diffractograms of PA11 and PA11-SGW composites.

The crystalline structure of PA11 and PA11-SGW composites was analysed using an X-ray diffractometer (Figure 5). It is known that PA11 shows polymorphism that highly influences its properties [41,42]. The DSC study showed two different structures during the second melting (γ and α’ forms), for PA11 and the composite with 10% of SGW. However, it must be noted that these structures were obtained after a melting process and controlled crystallization with a cooling rate of 10 °C/min. The obtained samples produced by injection-moulding were not cooled under the same conditions and can have a different structure. As can be seen in Figure 5, the samples after injection-moulding process showed a broad peak at 2θ = 21°, corresponding to the δ’ phase produced from quenching from the melt [35,43,44], which is similar to the process produced during injection-moulding. The controlled crystallization of the DSC led to obtaining the α’ crystalline form that was produced by melt crystallization [44]. Moreover, a small content of γ phase is typically obtained during this analysis and is transformed to the α’ more stable form [21]. Nonetheless, the γ phase or was impossible to be observed at room temperature [43] or its content was low and was difficult to identify. Thus, its formation simultaneously with the δ’ phase was not observed in the X-ray diffractograms.

The broad peak of PA11 and composites related with the PA11 δ’ crystalline form was similar to that obtained in the literature for this PA11 structure [17]. Despite this, two more peaks at 22.3° and 15° of 2θ appeared for PA11 + 20% SGW and PA11 + 50% SGW composites and were related with cellulose [29]. These peaks can only be appreciated in the composites with higher fibre contents due to the low crystallinity of the fibre: 48.5% measured with the X-ray diffractometer and calculated as the ratio between the intensity at the peak at 22°–23° and the minimum at 15°–18°, as has been described in the literature [45]. This low crystallinity of the SGW fibres is in agreement with the values obtained for untreated pine fibres [29]. These results differ from those found on the literature for PA11 nanocomposites, where, usually, the α’ structure is observed [21,36,44]. However, the reinforcement content with respect to the matrix for these nanocomposites was significantly lower. Moreover, the formation of the different phases is strongly influenced by the cooling process, which could be different than the performed in this work [35,44]. 

**Figure 6:**
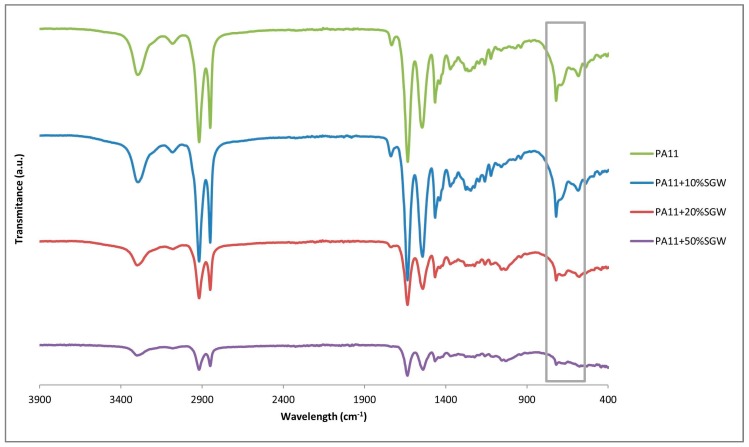
FT-IR PA11 and PA11-SGW composites.

Nonetheless, although γ phase is difficult to identify in the diffractograms at room temperature, FT-IR can serve as a complementary technique to determine the presence of this crystalline phase [39,45]. Figure 6 shows the FT-IR normalized for PA11 and PA11-SGW composites. There are some differences in the main bands observed regarding the crystal phase. Nevertheless, usually the difference in the wavelength is really small. One of the most differentiated bands in the FT-IR profiles was in the fingerprint zone of the FT-IR spectrum. The bands comprised in the range 500–800 cm^−1^ were related with amide V and VI amide bands (marked in the Figure 6). When the γ phase was present, a shoulder peak was observed in the 721 cm^−1^ peak, while in other forms a double peak at 721 and 686 cm^−1^ was shown. In PA11 and PA11 + 10% SGW a broad peak was observed while a double peak 721 and 681 cm^−1^ was appreciated in 20% and 50% of SGW fibre reinforced composites. Moreover, a small peak was observed around 627 cm^−1^ in the FT-IR of the PA11 and PA11 + 10% SGW composites and the band of 581 shifted to higher wavelengths, usually found in in the γ phase [45]. 

### 3.3. Effect of Annealing on the Structure and Thermal Transitions

Thermal annealing treatment is used in polymers to obtain a specific crystallinity form, to increase their crystallinity reducing the softening effect when the *T*_g_ is overpassed or as a useful form to remove residual stresses, which could appear during the extrusion, injection or other processing procedures [17,42,46]. Thermal annealing has growing importance in PA11 thermal studies because of its piezoelectric and ferroelectric properties. However, these studies usually involved the use of nanomaterials in the case of composite materials [21,34]. 

In this case, the objective of the annealing was to enhance the crystallinity of the samples to study the influence of SGW fibres during this process. As mentioned above, no considerable increase of the crystallinity was obtained in the DSC of PA11-SGW composites while a nucleating effect of the fibre was observed for PP-SGW composites [23]. As mentioned above, using CNF at low reinforcement contents increased the crystallinity of PA11. Otherwise, high contents of fibre seemed to inhibit the crystalline production, probably due to the H-bonds established between the fibres and the matrix. Crystalline structures in PA11 have a large dependence of the H-bond orientation [32]. In the literature, it can be found that, after an annealing process, the PA11 structure shows higher crystallinity [17,35]. However, the fibres and their capacity to interact with PA11 can impact this process. 

As explained before, the PA11, PA11 + 10% SGW, PA11 + 20% SGW and PA11 + 50% SGW samples were annealed at 165 °C for 1 h and studied using DSC, DMTA and X-Ray diffraction.

The DSC results showed an increase of the degree of the crystallinity in the first heat by the effect of the annealing, which increased considerably when the fibre content was augmented. In particular, the crystallinity of PA11 after the annealing treatment increased from 26.7% to 28.9%, and, in the composites, from 26.4%, 26.4% and 27.2% to 27.5%, 30.7% and 40.5% for PA11 + 10% SGW, PA11 + 20% SGW and PA11 + 50% SGW, respectively. It seems that SGW fibres can act as nucleating agent during the annealing process. Trans-crystallinity process between fibres and matrix are highly dependent on the characteristics of the fibre, matrix and their interactions [47]. PA11 requires higher time and temperature than PP due to the high intermolecular interactions, which can be produced between PA11 and fibres, explaining the increments produced during the annealing. The highest crystallinity was observed for the highest fibre content, with a difference in the crystallinity up to 10%, compared with the neat annealed PA11. 

**Figure 7:**
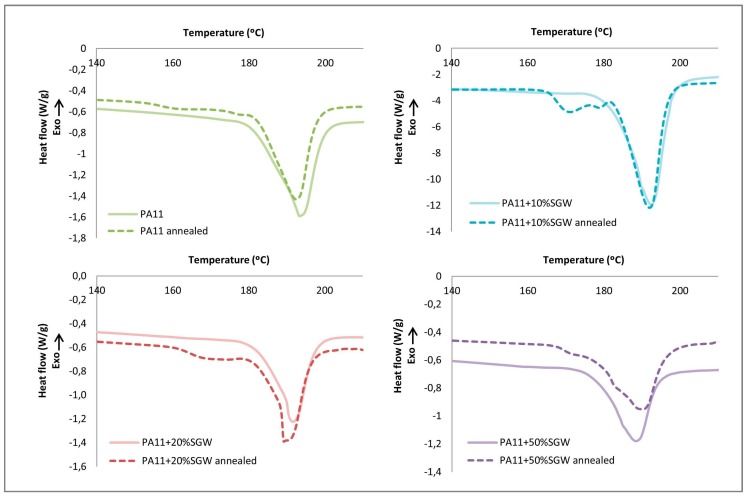
DSC thermographs of the firsts heating comparing annealed and not annealed samples.

Furthermore, a secondary crystal formation was observed in all the samples after annealing (Figure 7). PA11 showed a small peak around the temperature of the annealing treatment (165 °C) corresponding to the polymer chains crystallized during the annealing. In the composites, this small peak increased and was shifted to higher temperatures, indicating higher *T*_m_ of the crystals produced during the annealing process. In the case of PA11 + 50% SGW, the secondary crystal formation merged with the peak of the melting temperature at 189 °C. This phenomenon can be related with a nucleating effect of the fibre which has been observed in other polymers [22]. Moreover, the enhancement of the *T*_m_ of the crystals obtained after the annealing was also observed in the literature when the annealing time was increased for PA11 matrix [31]. The addition of SGW shortened the annealing treatment to achieve similar crystallinities.

Although an improvement in the crystallinity due the secondary crystal growth obtained during annealing was observed, only slight differences in the *T*_m_ values were found by the effect of the annealing treatment.

A change in the *T*_g_ was expected, as this transition temperature is directly related with the amorphous phase in the material. After the annealing, the amorphous phase of the polymer matrix was reduced, thus the crystalline phase was increased, diminishing the mobility of the chains. This can imply a higher *T*_g_ temperature. DMTA was performed in the annealed PA11 and their composites and the results were compared to the untreated ones. 

**Figure 8:**
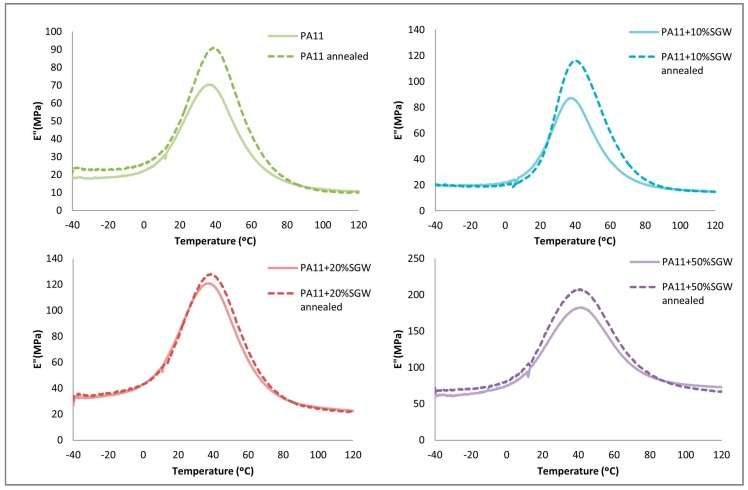
Loss modulus obtained by DMTA of the treated and untreated samples.

In the loss modulus (Figure 8), the annealed samples achieved higher values due to the higher crystalline phase in the polymer. Nonetheless, this increment in the modulus was reduced as the fibre content increased. Moreover, again, a unique transition corresponding to the *T*_g_ was observed in the studied range. However, the peaks were shifted to higher temperatures, except for the PA11 + 50% SGW. 

**Figure 9:**
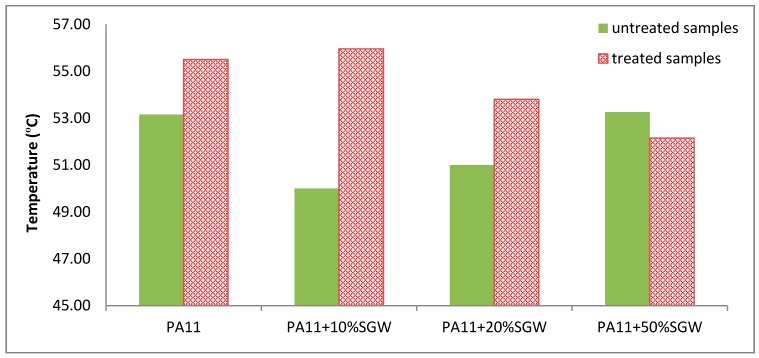
*T*_g_ measured values for treated and untreated samples.

As aforementioned, the tan δ was used to determine the *T*_g_ of the composite materials. Figure 9 shows the *T*_g_ obtained for untreated and treated samples.

The *T*_g_ temperatures increased for all the samples, except for the PA11 + 50% SGW, where similar *T*_g_ values were found for the untreated and annealed samples. On the other hand, the effect of the annealing in the *T*_g_ decreased as the fibre contents increased. This can be due to higher impact of the stiffness of the fibres in the modulus and in the reduction of the chain mobility than to the increase of crystallinity produced by the annealing treatment.

**Figure 10:**
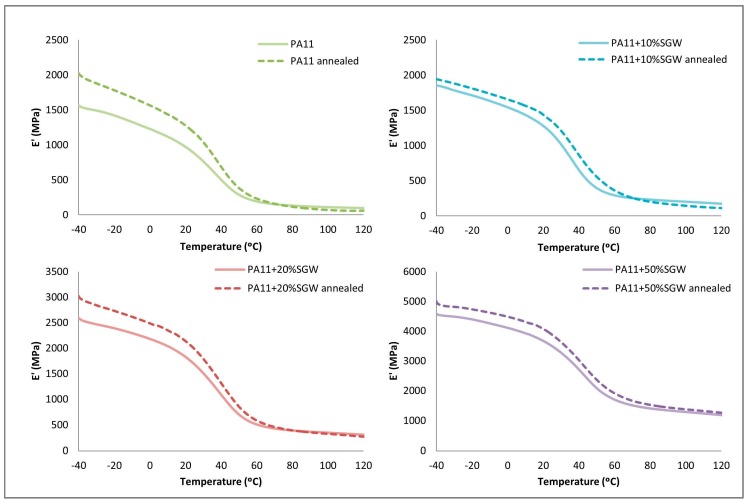
Storage modulus of treated and untreated samples obtained by DMTA.

Figure 10 shows the results obtained for the evolution of the storage modulus with the temperature analysed by DMTA. Higher moduli were obtained in the annealed samples due to the higher crystalline phase in the polymer. Nevertheless, as was observed, this effect was higher in the neat PA11 than in PA11-SGW composites.

**Figure 11:**
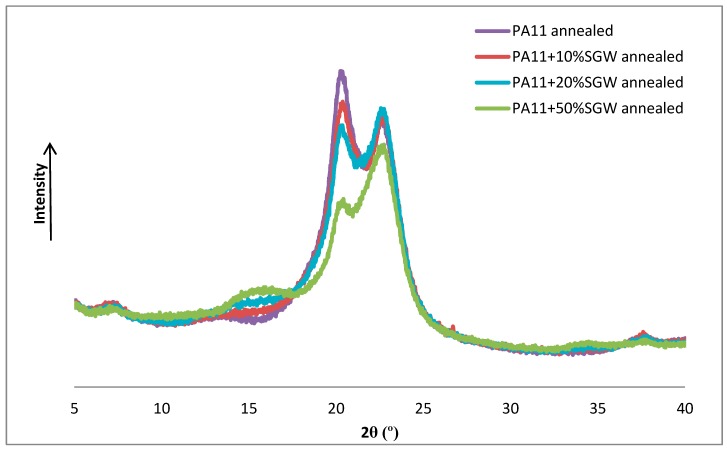
X-ray diffractograms of annealed samples.

Finally, the annealed samples were also studied by X-ray diffraction. As shown, the α’ form was observed in the neat PA11 and PA11-based composites (Figure 11). The annealing treatment produced a change in the polymer structure from the δ’ obtained after the injection-moulding process to the triclinic α’ structure, which is more thermodynamically stable [17,35]. The broad peak of the δ’ phase observed at 2θ = 21° was transformed in two well defined peaks at room temperature which shifted to 20.3° and 22.7°. The γ phase can be also obtained but is not usually observed under 100 °C [43]. 

**Figure 12:**
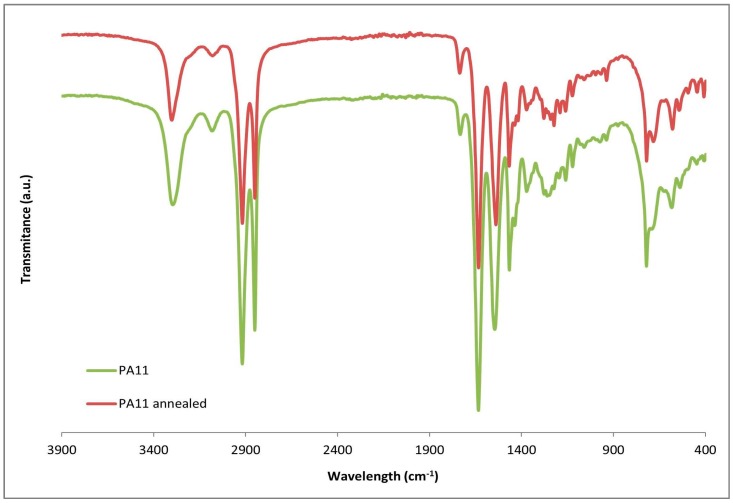
FT-IR of PA11 and annealed PA11.

Moreover, in the FT-IR of the PA11 annealed sample (Figure 12), the peaks shifted to α’ reported wavelengths [45] and their presence can be discarded or really reduced. In the composite materials, when the fibre content were augmented, the intensity of the second peak intensity corresponding to α’ form did the same, and exceeded the intensity of the first peak in the PA11 + 20% SGW and PA11 + 50% SGW composites. This effect, can be related with the overlap of this second peak with the cellulose peak and also with higher crystallinity [21] in the material which was in accordance with the obtained data in the DSC. 

The use of an annealing treatment has interest in lignocellulosic reinforced PA11 materials to increase the stiffness and the mechanical properties of the composite materials if working temperatures do not overpass *T*_g_. 

## 4. Conclusions

A thermal and structural characterization of PA11 and its composites reinforced with SGW was performed by means of TGA, DSC, DMTA and X-ray diffractionto determine the effect of the lignocellulosic reinforcement on the thermal transitions and morphology of the polymer matrix. A lower onset degradation temperature of PA11 by the effect of the fibres was found; however, once the decomposition temperature was started, the cellulosic fibres contributed to thermally stabilize the composites. No differences in *T*_m_ and *T*_c_, the main melting and crystallization peaks, and the crystallinity degree (26–27%) were found by the incorporation of SGW. Nonetheless, fibres promoted the α’ crystalline form instead of the both γ and α’ forms observed in the neat PA11. DMTA results revealed no considerable differences in the *T*_g_ of the composites with the fibre content attributed to the formation of an amorphous phase with more restricted mobility caused by the presence of the reinforcement. An improvement on the storage modulus was observed when increasing the fibre content throughout all the measured temperature range. 

Some samples were subjected to an annealing treatment during 1 h at 165 °C. The effect of the annealing on the materials was analysed by means of DSC and DMTA. The matrix crystallinity increased after the treatment. Moreover, higher crystal growth was also observed when the fibre content was augmented in the materials, achieving crystallinities increments around 13% for PA11 + 50% SGW. A slight increase of the *T*_g_ was observed, which can be related with the different crystal growths during the annealing treatment. A lower effect was obtained when the fibre content increased, probably due to the higher melting temperature of these crystals, similar to the PA11 melting point in the case of PA11 + 50% SGW composite. Slightly higher storage modulus values were obtained in the annealed samples, although the effect of the annealing in the stiffness seemed to decrease when the reinforcement content were increased. Finally, a different structure was observed before and after annealing in all samples. X-ray diffractograms showed a δ’ phase in the samples after the injection moulding process. However, in the annealed sample, this phase was transformed to a more stable α’ form. Moreover, FT-IR detected a small part of γ phase in neat PA11 and low fibre contents, which was not shown after the annealing. 

## Figures and Tables

**Table 1:** Onset temperatures for the 5% and 10% of weight loss and temperature in the maximum decomposition rate.

Temperatures (°C)	PA11	PA11 + 20% SGW	PA11 + 50% SGW
*T*_5%_	409	322	307
*T*_10%_	417	386	336
*T*_max_	439	451	461
